# Imaging and Quantification of Subbasal Nerve Plexus in Healthy Volunteers and Diabetic Patients with or without Retinopathy

**DOI:** 10.1371/journal.pone.0052157

**Published:** 2013-01-15

**Authors:** Andrey Zhivov, Karsten Winter, Marine Hovakimyan, Sabine Peschel, Volker Harder, Hans-Christof Schober, Guenther Kundt, Simone Baltrusch, Rudolf F. Guthoff, Oliver Stachs

**Affiliations:** 1 Department of Ophthalmology, University of Rostock, Rostock, Germany; 2 Translational Centre for Regenerative Medicine (TRM), University of Leipzig, Leipzig, Germany; 3 Clinic of Internal Medicine I, Klinikum Südstadt, Rostock, Germany; 4 Institute for Biostatistics and Informatics in Medicine and Ageing Research, University of Rostock, Rostock, Germany; 5 Institute of Medical Biochemistry and Molecular Biology, University of Rostock, Rostock, Germany; Charité University Medicine Berlin, Germany

## Abstract

**Background:**

The alterations of subbasal nerve plexus (SBP) innervation and corneal sensation were estimated non-invasively and compared with the values in healthy volunteers. Additionally, this study addressed the relation of SBP changes to the retinal status, glycemic control and diabetes duration.

**Methodology/Principal Findings:**

Eighteen eyes of diabetic patients with peripheral diabetic neuropathy aged 68.8±8.8 years and twenty eyes of healthy volunteers aged 66.3±13.3 yrs. were investigated with in vivo confocal laser-scanning microscopy (CLSM). An adapted algorithm for image analysis was used to quantify the morphological and topological properties of SBP. These properties were correlated to incidence of diabetic retinopathy (DR) and corneal sensation (Cochet-Bonnet esthesiometer). The developed algorithm allows a fully automated analysis of pre-segmented SBP structures. Altogether, 10 parameters were analysed, and all of them revealed significant differences between diabetic patients and healthy volunteers. The nerve fibre density, total fibre length and nerve branches were found to be significantly lower in patients with diabetes than those of control subjects (nerve fibre density 0.006±0.002 vs. 0.020±0.007 mm/mm^2^; total fibre length 6223±2419 vs. 19961±6553 µm; nerve branches 25.3±28.6 vs. 141.9±85.7 in healthy volunteers). Also the corneal sensation was significantly lower in diabetic group when compared to controls (43±11 vs. 59±18 mm). There was found no difference in SBP morphology or corneal sensation in the subgroups with (DR) or without (NDR) diabetic retinopathy.

**Conclusions/Significance:**

SBP parameters were significantly reduced in diabetic patients, compared to control group. Interestingly, the SBP impairment could be shown even in the diabetic patients without DR. Although automatic adapted image analysis simplifies the evaluation of in vivo CLSM data, image acquisition and quantitative analysis should be optimised for the everyday clinical practice.

## Introduction

The most common long-term complication of diabetes mellitus is diabetic neuropathy (DN) [1]. Population-based studies have reported prevalence for DN ranging from 8 to 54% in type 1 and from 13 to 46% in type 2 diabetes [2]. DN represents the third most common neurological disorder surpassed only by cerebrovascular events [3]. Most common type of DN is peripheral neuropathy, also called distal peripheral neuropathy (DPN), which represents 50% of diabetic neuropathy cases [4]. DPN affects nerve cells associated with the peripheral nervous system. DPN is primarily characterized by the loss of protective limb mechanical sensations. This can potentially lead to traumatic ulceration and amputation, primarily in the foot [5]. Moreover, DPN can progress to cardiac autonomic nerves, threatening mortality. Finally, about 11% of DPN cases are associated with chronic pain symptoms that severely diminish the quality of life and are frequently associated with depression [6].

DPN occurs due to preferential involvement of small nerve fibres (SNF) of the Aδ and C types [7]. Accurate assessment of small fibres involves a skin biopsy which is invasive, time consuming and requires significant laboratory expertise. Although this method has received a level “A” recommendation by the European Federation of Neurological Societies [8] and whilst the evaluation of intraepidermal nerve fibre density (IENFD) in skin biopsies has proven to be a useful method for the diagnosis of small fibre neuropathy, data on sensitivity, correlation with clinical symptoms and signs, and the diagnostic validity of IENFD in DN shows a relatively high degree of variability. The correlation between IENFD and neurophysiological function tests showed a wide range of findings in different studies [9].

The cornea is the most densely innervated part of the human body containing Aδ and unmyelinated C fibres derived from the ophthalmic division of the trigeminal nerve [10], [11]. Based on the evidence of impaired corneal sensitivity in diabetes [12] an increasing body of data indicates that a reduction in SBP fibres density and other morphological alterations in the cornea may be useful for the assessment of DPN [13]–[15].

Engineering of in vivo confocal laser-scanning microscopy (CLSM) in recent years has facilitated examination of the human cornea. This high resolution method has provided new insights on corneal nerve morphology and density in normal and pathological conditions with special emphasis on the subbasal nerve plexus-SBP [16]–[18]. The non-invasive nature of CLSM has the advantage of enabling examination of human cornea in its physiological state, avoiding artefacts induced by ex vivo histological techniques. This imaging modality allows multiple examinations of the same cornea over time. The in vivo technique developed by the authors and used for the corneal examination is the Heidelberg Retina Tomograph/Rostock Cornea Module (HRT/RCM).

Several studies have already shown the changes of SBP in case of diabetic neuropathy like decrease of nerve fibre length, density, tortuosity and number of branches[19]–[24]. Most of the studies performed with in vivo CLSM addressed the correlation of SBP morphology to the severity of DPN [22], [25]–[27]. Only few studies evaluated SBP with its possible relation to diabetic retinopathy (DR), which represents a major ocular complication of diabetes [23], [28].

DR affects the microvascular system in the patient’s eyes and is one of the main causes of blindness in the western world. Recently, the overall prevalence of DR in diabetic patients has been shown to be 29.2% [29]. The majority of these, approximately 80%, did not have sight-threatening DR (i.e. proliferative DR and/or maculopathy). Type 2 diabetic patients could have the background retinopathy at the time of the diagnosis of DM and about 60% of the patients develop any form of DR in 20 years [30].

It is not clear until now, whether DR as a microvascular complication is associated with small- or large-fibre neuropathy. The diabetic patients with neuropathy are more likely to have retinopathy as the ones without DN independent of age, sex, hypertension or lipid-lowering medication [31]. The multivariative risk analysis showed that the severity of DR is one of the main covariates for severity of DN.

On the basis of research literature mentioned above, the current study was designed to find out the possible relation between SBP morphology and DR.

## Materials and Methods

### Patients

This study was conducted after a positive appraisal by the ethics review board of Rostock University (permit number: A 2008 81). The research adhered to the tenets of the Declaration of Helsinki. A detailed consent review was carried out and the participants’ informed consent was obtained in writing.

Eighteen Type 2 diabetic patients aged 68.8±8.8 years (9 males/9 females, duration of diabetes 15.1±9.9 years, Hba1c 8.2±1.6) were recruited from the patients pool of the Clinic of Internal Medicine I, Rostock (the clinical data are given in Table 1). Patients with DN (NSS and NDS - mild till severe (i.e. 3–10 points)) and without other neuropathies were enrolled in the study.

**Table 1:** Clinical and demographic data of participants (data presented as mean ± SD).

Parameter	healthy volunteers (n = 20)	diabetic patients with neuropathy (n = 18)	diabetic neuropathy, DR (n = 7)	diabetic neuropathy, NDR (n = 11)
Age (yrs)	66.3±13.3	68.8±8.8	66.4±8.7	70.4±9.0
Gender (male/female)	10/10	9/9	3/4	4/7
Duration of DM (yrs.)	-	15.1±9.9	31.9±9.8	9.4±6.2
Hba1c (%)	-	8.2±1.6	9.2±1.1	7.6±1.6
Corneal sensation (mm)	59±18	43±11	41±7	45±13
neurologic symptom score, NSS (pts)	0.2±0.6	7.1±2.1	8.0±2.0	6.5±2.2
neurologic disability score, NDS (pts)	0.2±0.6	7.1±2.6	7.7±2.7	6.7±2.6

The patients with diabetes were further classified into 2 groups, based on the presence (DR) or absence (NDR) of diabetic retinopathy (DR), as assessed by ophthalmoscopy and seven-standard field stereoscopic 30° fundus photography (Nikon NF505, Nikon Corporation, Tokyo, Japan) (Table 2).

**Table 2:** Clinical data of individual patients in diabetes group (data presented as mean ± SD).

Patients	Diabetes duration	DR/NDR	NSS	NDS	HbA1c
1	12	NDR	7	4	8.7
2	24	DR	10	10	8.7
3	6	NDR	4	4	11
4	24	DR	7	8	8.4
5	28	DR	5	6	9.9
6	22	DR	10	8	11.2
7	14	NDR	9	9	5.5
8	6	NDR	9	8	7.3
9	37	DR	7	8	8.7
10	18	DR	7	10	7.9
11	1	NDR	8	9	7.1
12	9	NDR	8	8	7.6
13	3	NDR	7	6	7.2
14	11	NDR	4	3	7.2
15	7	NDR	8	9	5.2
16	27	DR	10	8	9.7
17	10	NDR	3	4	8.8
18	14	NDR	5	10	7.6

The control group included 20 healthy volunteers (10 males/10 females, aged 66.3±13.3 years) recruited from the patients’ pool of the Department of Ophthalmology, University of Rostock. The inclusion criteria for both groups were absence of current or previous local or systemic disease that could affect the cornea, and a negative history of ocular infections, eye surgery, trauma, allergy or use of contact lenses.

### Corneal Sensation

Corneal esthesiometry was obtained using the Cochet-Bonnet esthesiometer (Luneau Ophthalmologie, France). The nylon monofilament had a diameter of 0.12 mm, which dependent on the length exerted more or less pressure. The fully extended length of nylon filament was 60 mm. The central cornea was touched three times, beginning at a filament length of 60 mm. If a positive answer was not detected the filament length was shortened in steps of 5 mm each time and the procedure was repeated until there was a positive response.

### In Vivo Confocal Laser-scanning Microscopy (CLSM)

The corneas of healthy volunteers and diabetic patients were investigated in vivo by the HRT II in combination with the Rostock Cornea Module-RCM (Heidelberg Engineering, Heidelberg, Germany). The HRTII/RCM system uses as a laser source a diode laser with a wavelength of 670 nm and is equipped with a water contact objective (63×/0.95W, 670 nm; Zeiss, Jena, Germany). The distance from the cornea to the microscope is kept stable by a single-use contact element in sterile packaging (Tomo-Cap). Coupling between the patient’s cornea and the cap is facilitated with a thin lubricant layer of Vidisic gel (Bausch & Lomb/Dr. Mann Pharma, Berlin, Germany; refractive index 1.35). The eye to be examined was anesthetised by instilling Proparakain 0.5% eye drops (Ursapharm, Saarbrücken, Germany).

Image acquisition of the central cornea was performed in z-scan of automatic volume scan mode (30 images, volume depth 60 µm, constant interslice distance 2 µm). The acquired images have a definition of 384 x 384 pixels over an area of 400 µm^2^.

Confocal microscopy was performed in the region of interest, i.e. at the level of basal cells, SBP, Bowman’s membrane and anterior stroma at depths from 30 to 90 µm. At least three scans were performed. The total duration of microscopy was about 15 minutes.

### Quantification of Micromorphological Parameters

The evaluation of SBP on the basis of best artefact free single image was performed automatically using in house developed software tool [32] and was based on morphological (length, diameter, density) and topological (continuity and connectivity) parameters. For the latter the networks of nerve fibres were transformed into connected graphs consisting of paths and different types of nodes in order to quantify these graphs in the sense of a context-free measure of connectedness. We analysed images that were already segmented using GIMP 2.8.2 (www.gimp.org) manually, so automated image segmentation was not necessary. We called these images "pre-segmented". Image analysis was carried out in two stages. Firstly the segmented images were analysed morphologically and after this step the fibres were skeletonised in order to obtain their one pixel wide medial axis. In a second stage the topological analysis of the medial axis network took place. Consequently the main parameters were split up into two main categories: (1) before skeletonisation and (2) after skeletonisation (Table 3). Some of the parameters can also be computed in relation to the number of connected components (individual nerve fibre networks within the image area). All parameters of the measurements can be used isolated, combined or weighted for quantification of the SBP-networks.

**Table 3:** Parameters of automatic quantification of SBP structures.

**(1) before skeletonisation**	
component pixels (n)	number of pixels identified as nerve fibres
component ratio (%)	percentual covering of nerve fibres in respect to the image area
homogeneity of component pixel	a measure for the distributional uniformity of component pixels
**(2) after skeletonisation**	
skeleton pixels (n)	number of pixels representing the medial axes of nerve fibres
single nerve fibres (n)	number of all nerve segments between nerve branches and nerve ends
total nerve fibre length/averagesingle nerve fibre length (µm)	length of all nerve fibres in the image area/average length of single nerve fibres
nerve fibre density (mm/mm^2^)	length of all fibres in respect to the image area
number of branches (n)	number of branches
number of connectivity points (n)	the number of nerve fibres entering or leaving the image area to the outside
number of endpoints (n)	the number of nerve endings

### Statistical Analysis

All data were stored and analysed using the SPSS statistical package 17.0 (SPSS Inc. Chicago, Illinois, USA). Descriptive statistics were computed for continuous and categorical variables. The statistics computed included mean and standard deviations of continuous variables, frequencies and relative frequencies of categorical factors. Differences in continuous variables between the groups were investigated using the independent *t*-test or Mann-Whitney test, as appropriate. Test selection was based on an evaluation of variables of differences for normal distribution using the Kolmogorov-Smirnov test. Discriminant analysis was applied in order to identify a linear combination of most useful and sufficient quantitative predictor variables which best characterise the differences among the groups. Each predictor value was entered or removed step by step, Wilkś lambda as a multivariate analysis of variance test statistic was used. All *p* values resulted from two-sided statistical tests and values of *p*<0.05 were considered to be statistically significant.

## Results

The demographic and clinical data of healthy volunteers and diabetic patients are presented in Table 1.

The mean age and sex ratio of the control subjects were not significantly different from those of the patients with diabetes. Additionally, the diabetic patients were stratified in 2 subgroups: with (DR, n = 7) and without (NDR, n = 11) diabetic retinopathy. These both subgroups did not differ in age (*p* = 0.37), but in duration of diabetes (*p* = 0.004) and Hba1c (*p* = 0.01).

NSS and NDS scores did not differ in DR and NDR subgroups (*p* = 0.17 and *p* = 0.45 respectively) but were significantly increased compared to controls (both *p*<0.001). Corneal sensation was 59±18 mm in healthy volunteers and 43±11 mm in diabetic patients (*p*<0.001) (Table 1). The corneal sensation was not significantly different between DR and NDR patients (*p* = 0.43).

The DN group was characterised with an NSS of 7.1±2.1 and NDS of 7.1±2.6 pts. vs. 0.2±0.6 pts. for both NDS and NSS in control group (both *p*<0.001). There were no significant differences between the DR and NDR patients regarding NSS and NDS (*p* = 0.17 and *p* = 0,45).

The individual clinical data of diabetic patients are given in the Table 2. Here, it becomes obvious, that the incidence of diabetic retinopathy (DR) increases with the disease duration and correlates in most of the patients with a high HbA1c value.

**Figure 1:**
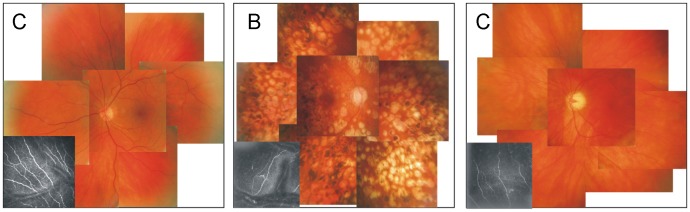
Seven standard-field fundus photography with corresponding CLSM images. Regular SBP pattern and normal fundus appearance in control (A). Patches of retinal whitening and rarefication of nerve fibers in SBP in a patient with DR (B). Relatively impaired appearance of SBP in a patient without DR (C).

In vivo CLSM revealed alterations in SBP morphology between control subjects and diabetic patients. Qualitatively, it seemed to be a reduction in nerve fibre and branch density in diabetic patients (Fig. 1B, C), compared to controls (Fig. 1A). In vivo CLSM also visualised a slight reduction of nerve fibre density in diabetic patients with retinopathy (DR) compared to those without retinopathy (NDR) (Fig. 1, B and C respectively).

**Figure 2:**
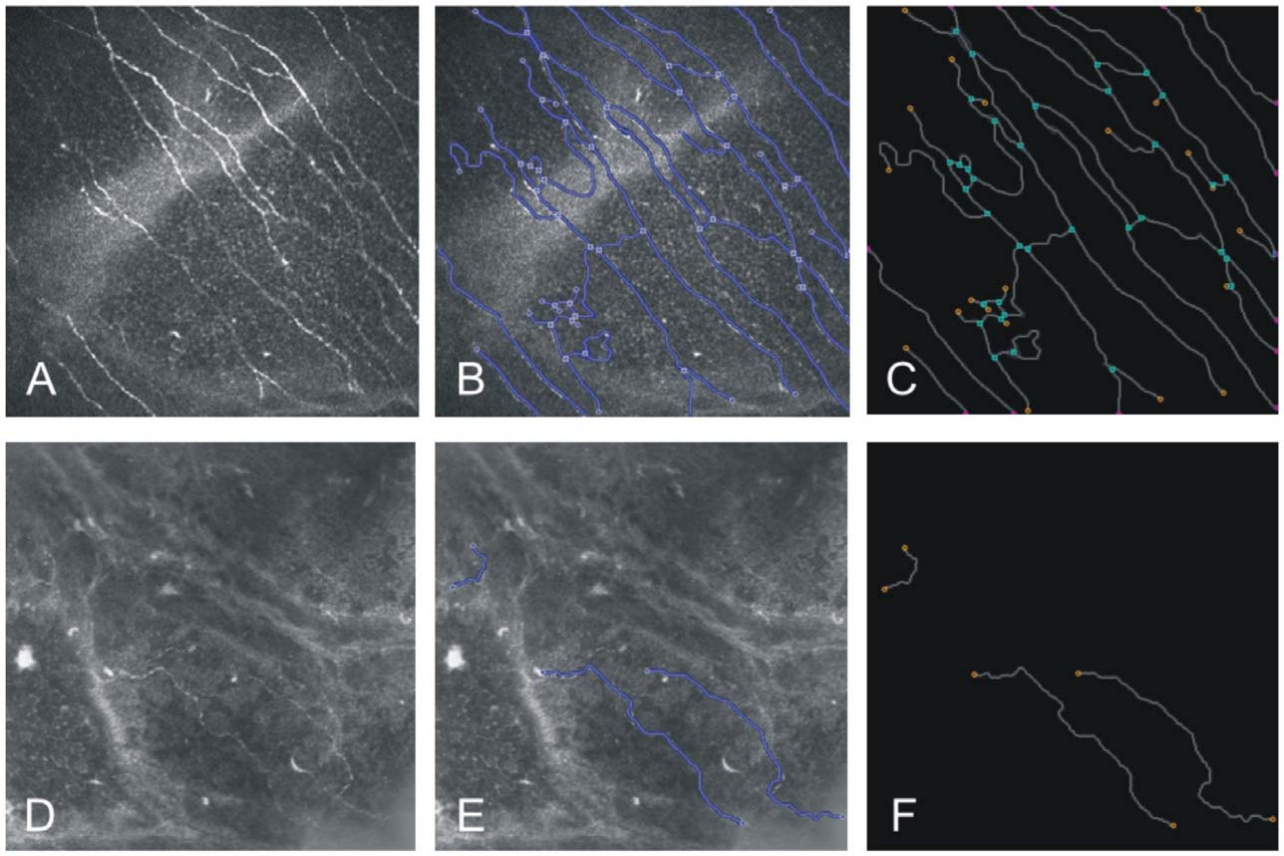
Confocal microscopy and quantification of micromorphological parameters. Initial image of SBP in the central cornea obtained with in vivo CLSM in a healthy volunteer (A) with corneal sensation 60 mm and diabetic patient (D) with corneal sensation 40 mm and NDS = 8 (image size: 400 x 400 µm). B and E represent the results of segmentation from the corresponding SBP images in control and diabetic subjects, respectively. C and F show graphs displaying the geometry of SBP in a final surface reconstruction. Total fibre length of 4706 and 545.4 µm, nerve fibre density 0.034 mm/mm^2^ and 0.004 mm/mm^2^, and single nerve fibre count 68 and 3 were measured in control subject and diabetic patient, respectively.

To verify these qualitative findings a further detailed image analysis was conducted. Figure 2 shows exemplary images of SBP in a healthy volunteer (A-C) and a patient with diabetes type 2 (D-F).

The SBP structures in healthy patients displayed a typical pattern with hyperreflective nerve fibers running towards center, regular tortuosity and nerve branching (A). Comparatively, the patient with diabetes less nerve fibres with increased tortuosity and decreased reflectivity (D).

The difference between normal and pathological morphology of SBP becomes more evident in segmented (B and E) and in final reconstructed images (C and F).

In order to illustrate the most representative data the number of analysed parameters were reduced to these with significant differences between diabetic patients and healthy volunteers (*p*<0.05) That means the number of component pixels, nerve fibre components, skeleton pixels, single nerve fibres per component, total fibre length, average single fibre length, nerve fibre density, connectivity points, number of branches and homogeneity of component pixels was evaluated. These parameters are shown in Table 3.

The quantification of SBP (Table 4) showed significantly less structures of SBP in diabetic patients (DR and NDR as a one group) comparing to the controls: component pixels (*p*<0.001), skeleton pixels (*p<*0.001), component ratio (*p*<0.001), single nerve fibres (*p*<0.001), single nerve fibres per component (*p*<0.001), total fibre length (*p<*0.001), nerve fibre density (*p*<0.001), connectivity points (*p*<0.001), number of branches (*p*<0.001), homogeneity of component pixels (*p* = 0.001) and not significant reduction of average single fibre length (*p* = 0.08),

**Table 4:** Results of automatic quantification of SBP structures (data presented as mean ± SD).

parameter	healthy volunteers (n = 20)	diabetic patients with neuropathy (n = 18)	diabetic neuropathy, DR (n = 7)	diabetic neuropathy, NDR (n = 11)
component pixels [n]	52863.8±19766	21756.6±8363.2	18114.3±6091.1	24074.4±9024.8
skeleton pixels [n]	16469.7±5430.3	5223.6±2060.1	4325.9±1530.1	5794.9±2210.6
component ratio (%)	5.7±2.1	2.4±0.9	2±0.7	2.6±1
single nerve fibres [n]	302.2±148.9	78.1±53.2	68.8±40.3	84.1±61.1
single nerve fibres per component [n]	8.8±6.5	3.4±3.5	3.1±3.9	3.5±3.4
total fibre length [µm]	19961.3±6552.9	6223.1±2419.2	5171.2±1842.8	6892.4±2577.7
average single fibre length [µm]	74.8±26	95.8±42	91.3±53.3	98.7±35.6
fibre density [mm/mm^2^]	0.02±0.007	0.006±0.002	0.005±0.002	0.007±0.003
connectivity points [n]	21.8±9.9	5.8±5.8	3.6±2.4	7.3±6.9
nerve branches [n]	141.9±85.7	25.3±28.6	20.5±25.2	28.4±31.3
homogeneighty component pixels	0.009±0.004	0.014±0.005	0.017±0.006	0.013±0.004

All parameters are normalised to the area of 1 mm^2^.

The micromorphology of SBP did not show any significant difference between DR (n = 7) and NDR (n = 11): component pixels (*p* = 0.15), skeleton pixels (*p* = 0.15), component ratio (*p* = 0.15), single nerve fibres (*p* = 0.72), single nerve fibres per component (*p* = 0.54), total fibre length (*p* = 0.15), average single fibre length (*p* = 0.38), nerve fibre density (*p* = 0.15), connectivity points (*p* = 0.25), number of branches (*p* = 0.48), homogeneity of component pixels (*p* = 0.10).

Comparing the results of quantification of both subgroups (DR and NDR) with controls revealed the same significant changes as the whole group with diabetic patients.

Analysis of 10 variables (all parameters of Table 4 without homogeneity of component pixels) in multivariate test (Wilkś lambda) showed highly significant difference between the centroids (vector of means) of group of diabetic patients and controls (10 variables simultaneously) (*p*<0.001). Overall 95% (36 out of 38) of the cases are assigned to the correct group (100% in diabetic patients and 90% (18/20) in healthy volunteers). The same significant difference for separation between the two groups was achieved using only the variables component ratio and nerve fibre density: in total 95% (36 out of 38 cases) were correctly classified with 90% (18 out of 20) in control group and 100% in diabetic group.

## Discussion

The impact of diabetes mellitus on the eye is greater than of any other systemic disease. It is of great clinical importance to clarify how the ocular markers of diabetes correlate with each other and with neuropathy status.

Diabetic retinopathy (DR) is one of the most common complications of both type 1 and type 2 diabetes, affecting nearly all persons with 15 or more years of disease [33]. The incidence of DR has been proposed to be tightly linked to disease duration and glycemic control in both type 1 and type 2 diabetes [34]. In the present study we could confirm the correlation between diabetes duration and incidence of DR. Previous studies demonstrated that glycemic control has an important and persistent effect in reducing diabetic complications. It has been shown that tight glycemic control prevented the progression of the severity of DR [35]. Nevertheless, DR progresses in some patients despite good glycaemia control. Also, poor glycaemia control does not always lead to retinopathy [36]. In our cross-sectional study most of the patients with DR revealed a higher HbA1c level than patients without DR.

Like many other studies on retina in diabetes, we also focused in our study on microvascular changes. Nevertheless, the view of considering DR as a microvascular disease has been strongly argued, proposing that DR should not be regarded as a vascular pathology in isolation [37]. Neurodegeneration of retinal glial cells has been proposed as an underlying cause of retinal vascular changes [38]. One important target for neurodegeneration in the retina is ganglion cell layer, in which axonal loss and thinning of the retinal nerve fibre layer (RNFL) occur as consequences of apoptotic death of ganglion cells. Müller cells represent another potential target for programmed cell death; these are cells supporting vascular endothelium, and their apoptosis leads to dysfunction of small vessels and, consequently, to microangiopathy. Thus, there is a likelihood that the cellular neurodegenerative changes in the retina could precede microvascular changes, and serve as an earlier marker of DR. Recently, the relationship between RNFL thickness and DPN has been investigated in patients with diabetes type 2 by measuring the RNFL thickness using OCT and assessing DPN level using NDS score [34]. By comparison with controls, diabetic patients with NDS>5 revealed a significant thinning of RNFL. It should be noted, however, that RNFL thickness can vary depending on age, gender and ethnicity. Accordingly, to verify the hypothesis that neuronal abnormalities could precede vascular abnormalities and to consider the RNFL thickness an earlier marker of DR, population-based, age and gender-matched studies must be performed in future.

It is widely accepted that there is a positive correlation between the corneal sensation and SBP density in healthy and pathological cornea [39], [40]. In the present study the diabetic patients exhibited impaired structures of SBP associated with reduced corneal sensitivity, which was in line with previous studies in diabetes type 1 and 2 [19], [22]–[28], [41], [42]. However, only a few studies among these addressed the relationship between SBP morphology and DR [23], [28]. In our study, all patients, either with or without DR, revealed significant differences when compared to controls. Messmer and colleagues [23] failed to find any significant differences in corneal innervation between NDR and control group. This inconsistency certainly can be attributed to the different image processing and analysis techniques used [23], [28].

Currently, there is a general need to improve the image acquisition and the image processing techniques before the SBP can be used as a standard non-invasive accessible ophthalmic marker for early DPN in patients with diabetes and pre-diabetes.

Many studies on SBP morphology differ in their sampling and quantification processes, and the manner in which nerve density is defined, causing obvious difficulties when comparing the results from different studies. So far, it was not a surprise that evaluated results of 13 confocal studies published between 2001 and 2008 [43] stated a huge discrepancy concerning quantification parameters. One explanation for these variations is differences in image contrast between microscopes. Therefore, the comparison of quantitative parameters is only valid if the same confocal microscope has been used. Currently, there are 3 classes of in vivo confocal microscopes for corneal research: confocal laser scanning microscopes (CLSM), confocal slit-scanning microscopes (CSSM) and confocal tandem scanning microscopes (CTSM). The fine nerve branches of SBP are more visible with CLSM because of high contrast, as opposed to SSCM and TSCM which exhibit a decreased contrast towards the lateral edges of the image, leading to visualization of about 80% of existing nerve fibers in SBP. According to the recent literature, the in vivo CLSM (Heidelberg Retina Tomograph II or III equipped with Rostock Cornea Module) has become the most widely-used instrument for morphological assessment of SBP [40].

The trend towards quantitative studies has led to the development of a various approaches for characterisation of SBP parameters: (i) evaluation of nerve fibre density and length using Adobe Photoshop (Adobe Systems Incorporated, USA) [14] as well as using a accustomed automatic calliper [44], ImageJ (http://rsb.info.nih.gov/ij/) or NeuronJ as plug in for ImageJ; Analysis 3.1 (Soft Imaging System, Münster, Germany) [23], [45] or Scion (Scion Image for Windows, Scion Corporation, USA) [22]; (ii) analysis of nerve tortuosity through manual grading [14], [23] or implementing into MATLAB (MathWorks, USA) for tortuosity coefficient‘ evaluation [25], [45]. Additionally, we gathered our experience with Cell D (Olympus, Germany) and AMIRA (TGS Inc, USA) but the results were not satisfactory due to the high number of false positive and negative recognition phenomenon.

A number of fully automated systems for assessing SBP morphology have been developed recently [46]–[48], demonstrating a strong correlation between manual and fully automated methods. However, these algorithms are limited to a few parameters like nerve fibre length, density, branch density fibre width and intensity.

In our study an adapted image processing algorithm [32] was used to pre-process, segment and evaluate the morphological parameters of SBP. The analysis of the relative small group of patients revealed the possibility to detect the pathological findings based only on two parameters (component ratio and nerve density) with the same probability as using the whole list of parameters of our study.

We suppose that precise analysis of two-dimensional arrangement of nerve fibres in larger areas will allow reliable evaluation of SBP structures as well as improve our understanding of SBP damage pattern in case of peripheral neuropathy. So far, the next step of optimising image analysis is the microscopy of the large scale corneal areas. We have recently published the possibility of on-line two-dimensional mapping of the cornea with an image size of 3072×3072 pixels (3.2×3.2 mm) [49]. The current stage of our hard and software development allows the analysis of pre-segmented 3.2×3.2 mm image in about three hours using a standard office PC. Moreover, in order to minimize the artefact rate, we also suggested performing the pre-processing of confocal images using elastic registration with a following 3-dimensional reconstruction and extraction of artefact free nerve structures with subsequent segmentation of nerves [50]. Currently, the work is in progress to apply this advanced method to a large cohort of recently diagnosed type 2 diabetic subjects compared with an aged matched control group, stratified for DR and DPN status. The literature data concerning SBP damage to be directly correlated with the duration of diabetes are very controversial and inconsistent. We hope that by using our reliable analysis we will be able to show early SBP alteration before neuropathy manifestation.
